# Quantity and quality of nucleic acids extracted from archival formalin fixed paraffin embedded prostate biopsies

**DOI:** 10.1186/s12874-018-0628-1

**Published:** 2018-12-05

**Authors:** Jessica Carlsson, Sabina Davidsson, Jonna Fridfeldt, Francesca Giunchi, Valentina Fiano, Chiara Grasso, Renata Zelic, Lorenzo Richiardi, Ove Andrén, Andreas Pettersson, Michelangelo Fiorentino, Olof Akre

**Affiliations:** 1 Department of Urology, Faculty of Medicine and Health, University Hospital in Örebro, Örebro University, Södra Grevrosengatan, 70185 Örebro, Sweden; 2 Department of Pathology, F. Addari Institute of Oncology S. Orsola Hospital, Bologna, Italy; 3 Cancer Epidemiology Unit-CERMS, Department of Medical Sciences, University of Turin and CPO-Piemonte, Turin, Italy; 4 Clinical Epidemiology Unit, Department of Medicine Solna, Karolinska Institute, Stockholm, Sweden; 5 Department of Medicine Solna, Karolinska Institute, and Department of Urology, Karolinska University Hospital, Stockholm, Sweden

**Keywords:** Prostate biopsies, Archival formalin-fixed paraffin-embedded tissue, Nucleic acid isolation kits, RNA integrity number

## Abstract

**Background:**

In Sweden, human tissue samples obtained from diagnostic and surgical procedures have for decades been routinely stored in a formalin-fixed, paraffin-embedded, form. Through linkage with nationwide registers, these samples are available for molecular studies to identify biomarkers predicting mortality even in slow-progressing prostate cancer. However, tissue fixation causes modifications of nucleic acids, making it challenging to extract high-quality nucleic acids from formalin fixated tissues.

**Methods:**

In this study, the efficiency of five commercial nucleic acid extraction kits was compared on 30 prostate biopsies with normal histology, and the quantity and quality of the products were compared using spectrophotometry and Agilent’s BioAnalyzer. Student’s t-test’s and Bland-Altman analyses were performed in order to investigate differences in nucleic acid quantity and quality between the five kits. The best performing extraction kits were subsequently tested on an additional 84 prostate tumor tissues. A Spearman’s correlation test and linear regression analyses were performed in order to investigate the impact of tissue age and amount of tissue on nucleic acid quantity and quality.

**Results:**

Nucleic acids extracted with RNeasy® FFPE and QIAamp® DNA FFPE Tissue kit had the highest quantity and quality, and was used for extraction from 84 tumor tissues. Nucleic acids were successfully extracted from all biopsies, and the amount of tumor (in millimeter) was found to have the strongest association with quantity and quality of nucleic acids.

**Conclusions:**

To conclude, this study shows that the choice of nucleic acid extraction kit affects the quantity and quality of extracted products. Furthermore, we show that extraction of nucleic acids from archival formalin-fixed prostate biopsies is possible, allowing molecular studies to be performed on this valuable sample collection.

## Background

Prostate cancer (PCa) is the most common male cancer in Europe, with almost 400,000 new diagnoses each year [1]. The natural history of the disease is variable, ranging from slow-growing indolent tumors to highly aggressive lethal tumors. Even though currently used clinical factors for prostate cancer management (Gleason score, baseline PSA levels, clinical stage and tumor extent based on core biopsies) provide valuable information, there is a strong need to find prognostic biomarkers that identify patients in need of curative treatment among men with low and intermediate-risk disease.

Finding prognostic biomarkers for PCa in prospective studies requires large study populations with many years of follow-up to ensure appropriate end-point data. By conducting nested case-control studies in large repositories of archived prostatic tissue, the efficiency and cost-effectiveness of prognostic studies can be strongly improved, while preserving the validity of a prospective cohort study. In Sweden, virtually all human tissue samples obtained from diagnostic and surgical procedures have for decades been routinely stored in formalin-fixed, paraffin-embedded, (FFPE) form [2]. Through linkage with nationwide registers using the unique national registration number, this vast collection of samples are available for molecular epidemiological studies to identify biomarkers predicting mortality even in slow-progressing prostate cancer.

Nucleic acids of high quality are vital for downstream molecular applications, however fixation of tissues causes modifications of biomolecules such as cross-linkage of nucleic acids with proteins, covalent modifications of both DNA and RNA, and fragmentation of RNA, making it challenging to extract nucleic acids of high quality from FFPE tissues [3–5]. The quality of RNA is furthermore affected by several other parameters such as time from sample retrieval to fixation, duration and conditions of fixation, the paraffin embedding procedure, sample storage, and even the extraction protocol used [6–11]. Unfortunately, these pre-analytical procedures cannot easily be standardized between, or even within, laboratories, and researchers can only impact the choice of extraction procedure when performing studies on archival FFPE tissues. Numerous commercially available kits for extraction of nucleic acids from FFPE tissues exists on the market today, however there is no general consensus among scientists which extraction kit works best, as most protocols work well. Nevertheless, it has been shown that the quality of the extracted RNA can differ between extraction kits. Several studies have been performed comparing the performance of commercial extraction kits, and different or even discordant results have been reported by different groups [12–16].

In order to assess the ability of five commercially available kits to obtain high-quality DNA and RNA from FFPE tissues, we carried out four comparative trials on archival FFPE prostate needle biopsies. Furthermore, the best performing kits were used in order to assess the quantity and quality of nucleic acids extracted. The results from this study will subsequently be implemented in the Program for prediction of Mortality in prostate cancer (PROMORT) study, investigating prognostic biomarkers in prostate biopsy tissues (Zelic et al., submitted manuscript).

## Methods

### Study population

In this study, we included FFPE prostatic needle biopsy tissues from men who underwent prostate biopsies for suspicion of PCa at the Department of Urology, University hospital in Örebro, Sweden between 1992 and 2002. In the first part of the study, in order to assess the performance of five commercial kits for extraction of nucleic acids from FFPE biopsy tissues, we included 30 biopsies with normal prostatic histology. In the second part of the study, in order to assess the quantity and quality of the nucleic acids extracted from the FFPE biopsies, we included 84 biopsies with cancer. The study was approved by the Ethical committee in Stockholm, Sweden (Approval number 2012/1586–31/1).

### Assessment of nucleic acids extraction kits from FFPE biopsies with normal histology

The study material, consisting of 30 FFPE prostate biopsies with normal histology, was randomly divided into three different groups; 1) ten biopsies for comparison between the High Pure FFPE RNA Micro Kit (Roche Diagnostics, West Sussex, UK) and the RNeasy® FFPE kit (Qiagen, Hilden, Germany), 2) ten biopsies for comparison between the High Pure DNA FFPET Isolation Kit (Roche Diagnostics, West Sussex, UK) and the QIAamp® DNA FFPE Tissue kit (Qiagen, Hilden, Germany), and 3) ten biopsies for comparison of the best performing DNA and RNA kits from the two previous steps to the AllPrep® DNA/RNA FFPE kit (Qiagen, Hilden, Germany). In order to minimize the risk of variation due to different operators, the same operator performed all extractions with one kit (e.g. all extractions using the RNeasy® FFPE kit).

1. In the comparison between a) the High Pure FFPE RNA Micro kit and the RNeasy® FFPE kit and b) the High Pure FFPET DNA Isolation kit to the QIAamp® DNA FFPE Tissue kit, three serial sections of 10 μm were cut from ten biopsies with normal histology. In each case, the first section was discarded to exclude negative effects from exposure to air. The two remaining sections were placed on separate slides; tissues were scraped and put in separate Eppendorf tubes, and were subsequently used for RNA/DNA extraction using either one of the two different extraction kits being compared. The extraction procedure followed the manufacturers’ instructions. RNA was eluted with 20 μl elution buffer while DNA was eluted with 50 μl.
2. For comparison between the best performing RNA and DNA kits and the AllPrep® DNA/RNA FFPE kit, four serial sections of 10 μm were cut from the third set of ten biopsies with normal histology. In each case, the first section was discarded to exclude negative effects from exposure to air. Each subsequent section was placed on separate slides; tissues were scraped, put in separate Eppendorf tubes and were subsequently used for extraction of nucleic acids using the three different extraction kits. The extraction procedure followed the kit instructions and RNA/DNA was eluted using 20 μl and 50 μl of elution buffer, respectively.

### Assessment of DNA/RNA quantity and quality from FFPE biopsies with cancer

The study pathologists (F.G and M.F) assessed the prostatic biopsies for millimeter of tumor (tumor length), percentage of tumor tissue and Gleason score. They also circled tumor areas on the hematoxylin and eosin (H&E) slides corresponding to the tissue blocks. Three slices of 10 μm were subsequently sectioned from each biopsy tissue block; the first section was discarded while the remaining two sections from each case were used for macro-dissection of the tumor area using an RNase-free scalpel for each case. The macro-dissected tumor area from one tissue section was used for RNA isolation using the RNeasy® FFPE kit (Qiagen, Hilden, Germany) and the tumor area from the second section was used for DNA isolation using the QIAamp® DNA FFPE Tissue kit (Qiagen, Hilden, Germany), following the manufacturers’ instructions.

### Quantity and quality measurements

The quantity and purity (A260/A280) of the extracted DNA and RNA was measured using the NanoDrop ND-2000 Spectrophotometer (Thermo Scientific, Waltham, MA, USA). When using a spectrophotometer, an OD260/280 greater than 1.8 or 2.0 is generally considered to be an indicator of good RNA and DNA quality, respectively. Lower OD ratios could indicate the presence of contaminants, or very low concentrations (< 10 ng/μl) of nucleic acids in the sample. The RNA integrity was assessed using the Agilent 2100 BioAnalyzer and the Agilent RNA 6000 Pico kit (Agilent Technologies, Palo Alto, CA). The RNA Integrity Number (RIN) is a score of the RNA degradation ranging on a scale from 1 to 10, where 1 represents the most degraded RNA and 10 represents a completely intact RNA [17].

The endogenous control gene 18S rRNA is typically employed to allow quantitation of relative gene expression in DNA/RNA samples. In order to determine the presence of amplifiable 18S rRNA in the extracted samples, a qPCR was performed. Ten nanograms of total RNA were converted to cDNA using the SuperScript VILO master mix (Thermo Fisher Scientific, Waltham, MA, USA). The resulting cDNA or 10 ng of DNA from each sample were used in a qPCR reaction using a TaqMan probe for RNA18S5 (Hs03928985_g1) and TaqMan Fast Universal PCR Master Mix (Thermo Fisher Scientific, Waltham, MA, USA) and run in a 40-cycle reaction on the 7900 HT system (Applied Biosystems, Foster City, CA, USA). The threshold value was automatically calculated using the RQ Manager Version 1.2.1 software and C_T_-values of < 38 were considered positive.

### Statistical analyses

For assessment of the quality and quantity of the nucleic acids extracted with the five commercial extraction kits, both a paired Student’s t-test and a Wilcoxon matched-pairs signed rank test was performed in order to evaluate differences in yields, purity (A260/A280), RIN- and C_T_-values between the kits. A Bland-Altman analysis was performed in order to investigate the level of agreement of the two extraction kits. A fixed difference indicates that one method yields higher/lower values compared to the other method by a constant amount, while a proportional difference indicates if one method yields higher/lower values than those from the other method, which is proportional to the level of the measured variable [18].

In order to test if the percentage of tumor cells, length of the tumor tissue, or year of biopsy were associated with the quantity and quality of the extracted nucleic acids, a Spearman’s correlation test was performed. A multivariate linear regression models was performed in order to investigate associations between tumor length (mm), age of FFPE tissue, Gleason score and measurements of RNA/DNA quantity and quality. Tumor length and percentage of tumor cells was categorized and a Mann-Whitney U-test or a Kruskal-Wallis one-way analysis of variance was performed in order to test for differences in quantity and quality of nucleic acids between the categories. All statistical analyses were performed in SPSS Statistics version 22.

## Results

### Assessment of nucleic acids extraction kits

#### RNA extraction comparative trials

In the first trial, Roche Diagnostic’s High Pure FFPE RNA Micro kit was compared to Qiagen’s RNeasy® FFPE kit. The RNeasy® FFPE kit resulted in consistently higher RNA yields (mean yield 16.3 ng/μl) ranging from 8.1 ng/μl to 20.8 ng/μl compared to the samples extracted with High Pure FFPE kit (mean yield 5.6 ng/μl) ranging from 2.2 ng/μl to 11.2 ng/μl (*p* < 0.001). Furthermore, both the A260/A280 ratios and RIN-values in samples extracted with the RNeasy® FFPE kit were higher (mean A260/A280 = 1.55, mean RIN-value = 1.98) compared to samples extracted with the High Pure kit (mean A260/A80 = 1.38, mean RIN-value = 1.04) (*p* = 0.03 and *p* < 0.001, respectively). To evaluate the quality and efficiency of the extracted RNA, the expression levels of 18S5 rRNA was measured by qPCR. All samples were amplifiable as indicated by a C_T_-value < 38. Samples extracted with the RNeasy® FFPE kit yielded lower C_T_-values compared to samples extracted with the High Pure kit (mean C_T_ 25.4 versus 26.4), however this difference was not statistically significant (Table 1).

**Table 1:** Comparison of RNA quantity and quality isolated by three different commercially available kits

RNA extraction	Comparison 1			Comparison 2		
	High Pure	RNeasy®	*p*-value	AllPrep®	RNeasy®	*p*-value
FFPE tissue input	10 μm	10 μm		10 μm	10 μm	
Successful extractions	10/10	10/10		10/10	10/10	
RNA yield (ng/μl)						
Minimum	2.2	8.1		8.0	6.7	
Maximum	11.2	20.8		16.8	16.7	
Mean (95% CI)	5.6 (3.4;8.2)	16.3 (13.1;19.0)	< 0.001^a^	12.6 (10.8;14.6)	11.4 (9.1;13.8)	0.307^a^
SD	3.1	3.8		2.5	3.1	
A260/A280						
Minimum	1.23	1.41		1.39	1.39	
Maximum	1.62	1.69		1.59	1.57	
Mean (95% CI)	1.38 (1.28;1.48)	1.55 (1.51;1.61)	0.003^a^	1.50 (1.43;1.55)	1.47 (1.43;1.52)	0.419^a^
SD	0.13	0.06		0.07	0.06	
RIN-value						
Minimum	1.0	1.0		1.0	1.0	
Maximum	1.2	2.5		2.5	2.5	
Median	1.0	2.15	0.011^b^	1.0	2.5	0.044^b^
IQR	0.10	0.88		0.15	0.40	
C_T_-value RNA18S5						
Minimum	21.2	23.6		26.1	24.9	
Maximum	30.9	27.2		30.1	28.9	
Mean (95% CI)	26.4 (23.7;28.9)	25.4 (24.3;26.3)	0.268^a^	28.0 (27.1;28.9)	27.4 (26.5;28.4)	0.088^a^
SD	3.36	1.3		1.22	1.26	

Comparison of RNA quantity and quality isolated by three different commercially available kits, High Pure FFPE RNA Micro kit, RNeasy® FFPE kit and AllPrep® DNA/RNA FFPE kit. *SD* Standard deviation, *IQR* Inter Quartile Range. ^a^ Student’s paired T-test, and ^b^ Wilcoxon matched-pairs signed rank test

**Fig. 1:**
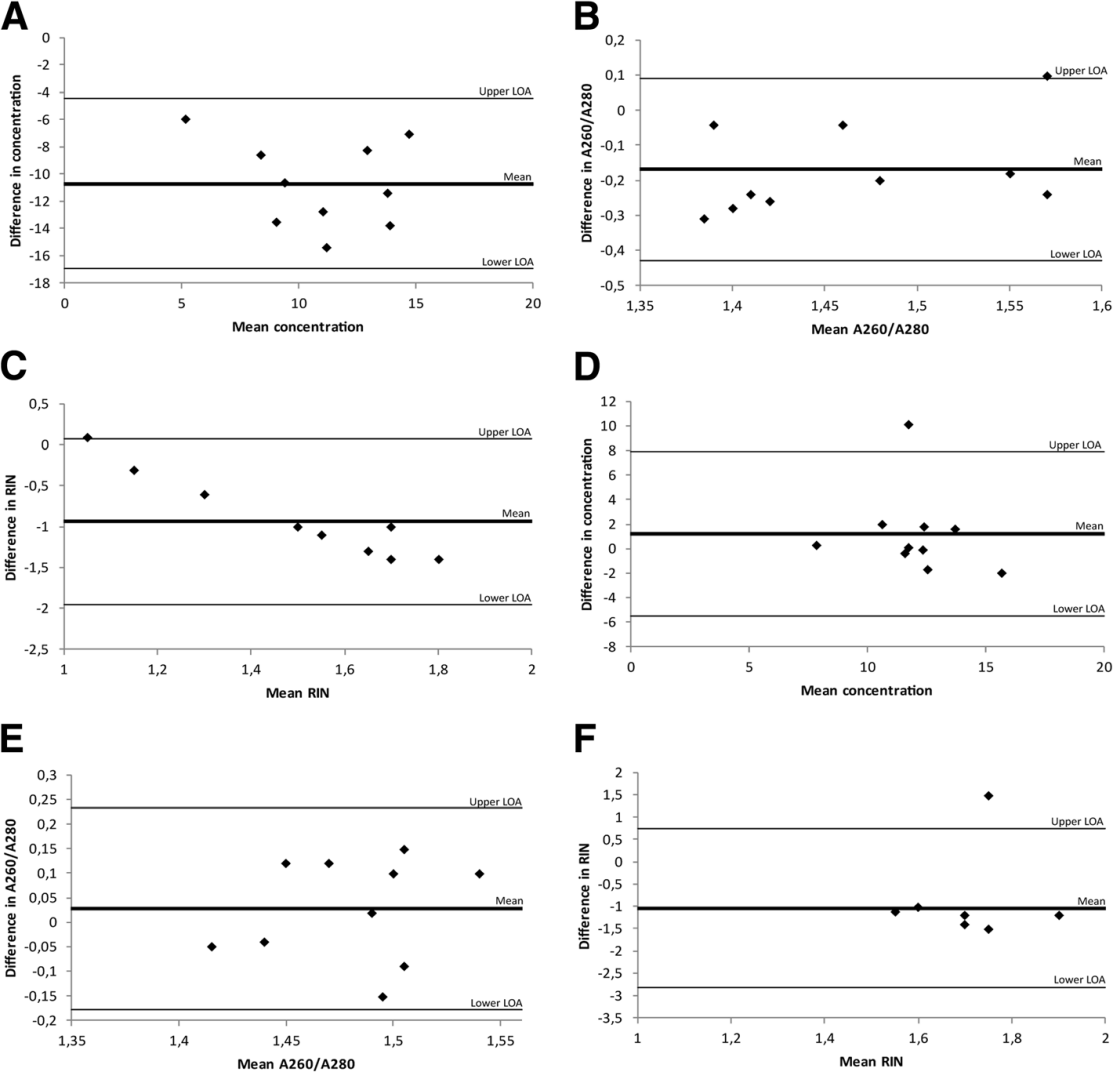
Bland-Altman plots for investigating the level of agreement between RNA extraction kits. Each plot shows the differences between the two kits against the averages of the two kits. The lines represent the mean differences and upper and lower limits of agreement (LOA, mean differences ±1.96SD). **a** Comparison of RNA yield (ng/μl) of samples extracted with High Pure FFPE RNA Micro Kit and RNeasy® FFPE kit. **b** Comparison of purity (A260/A280) of samples extracted with High Pure FFPE RNA Micro kit and RNeasy® FFPE kit. **c** Comparison of RIN-values of samples extracted with High Pure FFPE RNA Micro kit and RNeasy® FFPE kit. **d** Comparison of RNA yield (ng/μl) of samples extracted with RNeasy® FFPE kit and AllPrep® DNA/RNA FFPE kit. **e** Comparison of purity (A260/A280) of samples extracted with RNeasy® FFPE kit and AllPrep® DNA/RNA FFPE kit. **f** Comparison of RIN-values of samples extracted with RNeasy® FFPE kit and AllPrep® DNA/RNA FFPE kit

Figure 1a-c shows a comparison of RNA yields, purity (A260/A280) and RIN-values obtained from samples extracted with the High Pure FFPE RNA Micro kit and the RNeasy® FFPE kit, respectively. A fixed difference was indicated by a mean difference of − 10.71 (limits of agreement: − 16.96 – − 4.46) when investigating the agreement in yields between the two kits. This observation indicates that the High Pure kit consistently produced lower yields. A fixed difference was also seen for the purity of the extracted RNA (mean difference − 0.17; limits of agreement: − 0.42 – 0.09) and the RIN-values (mean difference − 0.94; limits of agreement: − 1.95 – 0.07). No proportional difference was evident for RNA yields (slope: 0.269, *p* = 0.48) or purity (slope: − 0.7, *p* = 0.255), although a proportional difference was evident for RIN-values (slope: 1.98, *p* < 0.0001).

In the second trial of comparisons, the RNeasy® FFPE kit was compared to the RNA fraction of the samples extracted with Qiagen’s AllPrep® DNA/RNA FFPE kit. There was no evidence of difference in yields (11.4 ng/μl versus 12.6 ng/μl), A260/A280 ratios (1.47 versus 1.50) or C_T_-values (27.4 versus 28.0) between these two kits, although higher RIN-values were seen for samples extracted with the RNeasy® FFPE kit (2.2 versus 1.2, *p* = 0.006)(Table 1). A Bland-Altman analysis indicated there were no fixed differences in yield (mean difference 1.17; limits of agreement: − 5.53 – 7.87) or A260/A280 ratios (mean difference 0.03; limits of agreement: − 0.18 – 0.23). However, a fixed difference was seen for RIN-values, with the AllPrep® DNA/RNA FFPE kit consistently producing lower RIN-values compared to samples extracted with the RNeasy® FFPE kit (mean difference − 1.04; limits of agreement: − 2.83 – 0.75). No proportional difference was evident for neither yield (slope: 0.318, *p* = 0.604), purity (slope: − 0.583, *p* = 0.567), or RIN-values (slope: − 0.099, *p* = 0.978) (Fig. 1d-f).

#### DNA extraction comparative trials

**Fig. 2:**
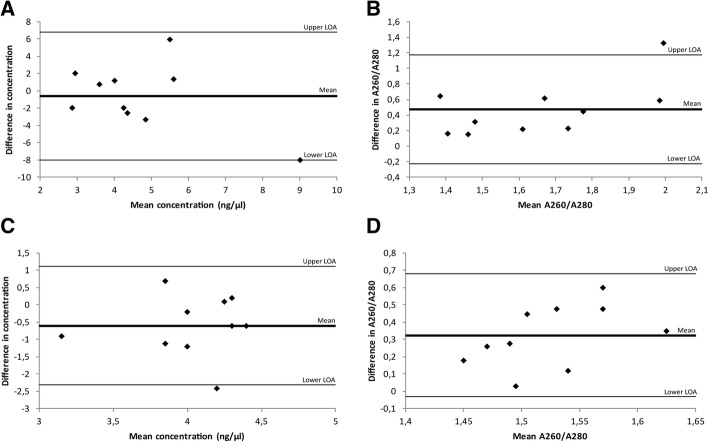
Bland-Altman plots for investigation of level of agreements between DNA extraction kits. Each plot shows the differences between the two kits against the averages of the two kits. The lines represent the mean differences and upper and lower limits of agreement (LOA, mean differences ±1.96SD). **a** Comparison of DNA yield (ng/μl) of samples extracted with High Pure FFPET DNA Isolation kit and QIAamp® DNA FFPE Tissue kit. **b** Comparison of purity (A260/A280) of DNA samples extracted with High Pure FFPET DNA Isolation kit and QIAamp® DNA FFPE Tissue kit. **c** Comparison of DNA yield (ng/μl) of samples extracted with QIAamp® DNA FFPE Tissue kit and AllPrep® DNA/RNA FFPE kit. **d** Comparison of purity (A260/A280) of samples extracted with QIAamp® DNA FFPE Tissue kit and AllPrep® DNA/RNA FFPE kit

In the third comparative trial, Qiagen’s QIAamp® DNA FFPE Tissue and Roche Diagnostic’s High Pure FFPET DNA Isolation kit was investigated. Both kits resulted in similar DNA yields (4.4 ng/μl versus 5.0 ng/μl). However, samples extracted with the QIAamp® FFPE kit had higher A260/A280 ratios compared to samples extracted with the High Pure DNA kit (mean ratio 1.89 versus 1.37, *p* = 0.002). To evaluate the quality and efficiency of the extracted DNA, a qPCR measuring the expression levels of 18S5 rRNA was performed, and all samples were amplifiable as indicated by a C_T_-value < 38. A difference in C_T_ –values could be seen for samples extracted with the two kits (mean C_T_ 28.6 versus 27.1, *p* = 0.011), with samples extracted with the QIAamp® kit yielding higher C_T_-values compared to samples extracted with the High Pure DNA kit (Table 2). A Bland-Altman analysis did not indicate a fixed difference for yield for the two kits (mean difference − 0.61; limits of agreement: − 8.03 – 6.81), however, a fixed difference was seen for A260/A280 ratios (mean difference 0.35; limits of agreement: − 0.22 – 1.17). No proportional difference was evident for either DNA yield (slope: − 1.019, *p* = 0.162) or purity (slope: 0.969, *p* = 0.061) (Fig. 2a-b).

**Table 2:** Comparison of DNA quantity and quality isolated by three commercially available kits

DNA extraction	Comparison 1			Comparison 2		
	High Pure	QIAamp®	*p*-value	AllPrep®	QIAamp®	*p*-value
FFPE tissue input	10 μm	10 μm		10 μm	10 μm	
Successful extractions	10/10	10/10		10/10	10/10	
DNA yield (ng/μl)						
Minimum	1.9	1.9		3.5	2.7	
Maximum	13.0	8.5		5.4	4.4	
Mean (95% CI)	5.0 (2.4;7.6)	4.4 (2.7;5.6)	0.623^a^	4.4 (3.9;4.7)	3.8 (3.3;4.2)	0.059^a^
SD	3.3	1.9		0.6	0.6	
A260/A280						
Minimum	1.06	1.49		1.27	1.51	
Maximum	1.69	2.66		1.48	1.87	
Mean (95% CI)	1.37 (1.27;1.57)	1.89 (1.58;2.17)	0.002^a^	1.36 (1.29;1.41)	1.68 (1.62;1.79)	< 0.001^a^
SD	0.19	0.38		0.07	0.12	
C_T_ -value RNA18S5						
Minimum	24.7	27.0		26.6	27.9	
Maximum	29.5	31.1		28.5	30.9	
Mean (95% CI)	27.1 (26.1;28.1)	28.6 (27.4;29.2)	0.011^a^	27.3 (26.9;27.9)	29.0 (28.2;30.1)	< 0.001^a^
SD	1.29	1.17		0.68	1.23	

Comparison of DNA quantity and quality isolated by three commercially available kits; High Pure FFPET DNA Isolation kit, QIAamp® DNA FFPE Tissue kit, and AllPrep® DNA/RNA FFPE kit. SD – Standard Deviation, CI – Confidence Interval. ^a^ Student’s paired T-test

In the fourth and final comparative trial, the QIAamp® DNA FFPE Tissue kit was compared to the DNA fraction from the AllPrep® DNA/RNA FFPE kit. There was no evidence of difference in DNA yield (mean yield 3.8 ng/μl versus 4.4 ng/μl) for samples extracted with the two kits, however higher A260/A280 ratios were seen in samples extracted with the QIAamp® kit (mean ratio 1.68 versus 1.36, *p* < 0.001). Furthermore, samples extracted with the AllPrep® kit yielded lower C_T_ –values (mean C_T_ 29.0 versus 27.3, *p* < 0.001) (Table 2). A Bland-Altman analysis revealed a fixed difference for A260/A280 ratios (mean difference 0.32; limits of agreement: − 0.03 – 0.68) but not for DNA yield (mean difference − 0.6; limits of agreement: − 2.32 – 1.12). No proportional difference was evident for either yield (slope: − 0.158, *p* = 0.856) or A260/A280 (slope: − 1.646, *p* = 0.158) (Fig. 2c-d).

### Assessment of DNA/RNA quantity and quality from archival biopsies

**Fig. 3:**
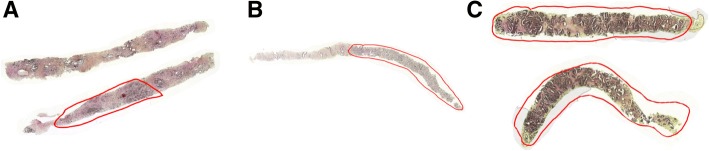
Examples of DNA/RNA yield from biopsies containing varying tumor length. Tumor tissue is indicated in red. **a** A tumor length of 3.4 mm yielded 4.4 ng/μl RNA and 2.8 ng/μl DNA. **b** A tumor length of 7.1 mm yielded 8.4 ng/μl RNA and 8.0 ng/μl DNA. **c** A tumor length of 12.8 mm yielded 26.3 ng/μl RNA and 12.5 ng/μl DNA

In this part of the study, 84 FFPE needle prostate biopsy tissues with malignant histology were included. Selected characteristics from the biopsies can be seen in Table 3. Based on the results from the assessment of nucleic acid extraction kits, DNA and RNA were extracted from two serial sections of the biopsies using the QIAamp® DNA FFPE Tissue Kit and RNeasy® FFPE kit. Both DNA and RNA were successfully extracted from all biopsies, with varying quantity and quality. The mean DNA yield for all 84 biopsies was 3.7 ng/μl (range: 0.6–12.5 ng/μl) and the mean A260/A280 ratio was 1.8 (1.1–5.3). The mean RNA yield was 10.6 ng/μl (1.6–30.9 ng/μl), mean A260/A280 was 1.5 (0.7–1.9) and the RIN-values ranged from 1.0–4.1, with a mean value of 2.3. Scanned images of biopsies from three patients and the resulting DNA and RNA yield/biopsy can be seen in Fig. 3. To evaluate the quality and efficiency of the extracted DNA and RNA, a qPCR measuring the expression levels of 18S5 rRNA was performed. One RNA sample and three DNA samples failed to reach the threshold of a C_T_-value < 38 (Table 4).

**Table 3:** Selected characteristics of malignant prostate biopsies

Characteristic	*N* = 84
Year of biopsy	1992–1998
Length of tumor, median mm (range)	4.3 (0.2–17.8)
% tumor cells (range)	40 (5–90)
Gleason score	
≤ 6	4
3 + 4	9
4 + 3	15
8–10	56

**Table 4:** Quantity and quality measurements of nucleic acids

Archival biopsies	RNA	DNA
FFPE tissue input	10 μm	10 μm
Number of extractions	84	84
Yield (ng/μl)		
Minimum	1.6	0.6
Maximum	30.9	12.5
Median	9.2	3.3
IQR	6.35	2.3
A260/A280		
Minimum	0.7	1.1
Maximum	1.9	5.3
Median	1.5	1.7
IQR	0.2	0.4
RIN-value		
Minimum	1.0	–
Maximum	4.1	–
Median	2.5	–
IQR	0.18	
C_T_ -value RNA18S5		
Minimum	22.0	24.6
Maximum	39.3	38.7
Mean	28.8	–
SD	3.1	–
Median	–	29.2
IQR	–	6.4

Quantity and quality measurements of nucleic acids isolated from archival FFPE biopsy tissues from the prostate tumor. *SD* Standard Deviation, *IQR* Inter Quartile Range

**Fig. 4:**
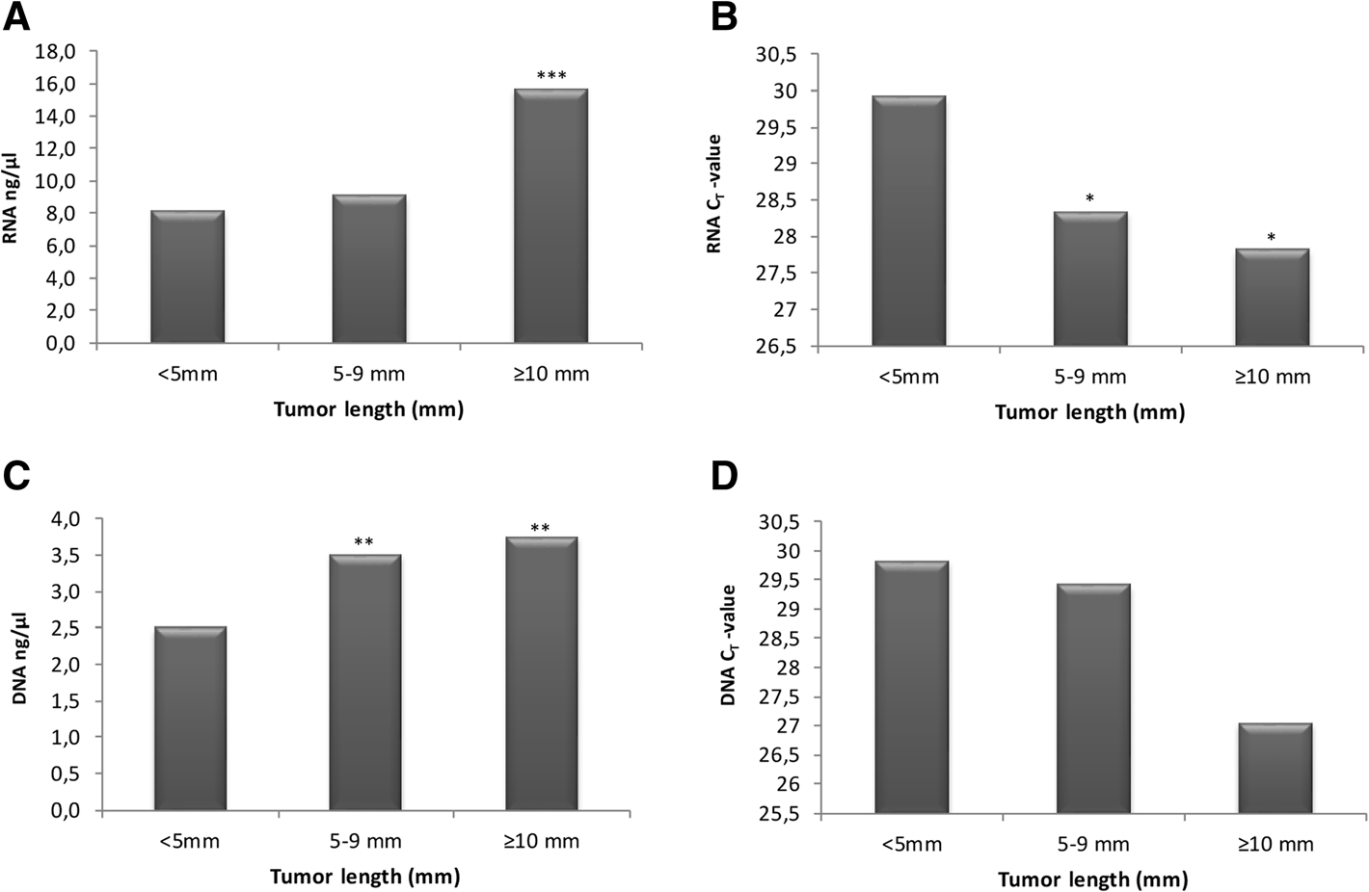
The effect of tumor length on DNA/RNA yield and RNA18S5 C_T_ -values. **a** RNA yield is increasing with increasing tumor length, (**b**) C_T_-values on RNA samples are decreasing with increasing tumor length, (**c**) DNA yield is increasing with increasing tumor length, and (**d**) C_T_-values on DNA samples are decreasing with increasing tumor length. * *p* < 0.05, ** *p* < 0.01, and ***p < 0.001 compared to < 5 mm cancer

There was evidence of correlation between tumor length (in mm) and RNA yield (*r* = 0.45, *p* < 0.001) and RIN-values (*r* = 0.37, *p* < 0.01) (Table 5), as well as for tumor length and DNA yield (*r* = 0.45, *p* < 0.001) (Table 6). When dividing tumor length into three categories (< 5 mm, 5–9 mm, and ≥ 10 mm), DNA/RNA yield as well as RNA18S5 C_T_-values differed among categories, with higher yields/lower C_T-_values with increasing tumor length (Fig. 4). No correlation between the DNA and RNA yield could be seen (*r* = 0.18, *p* = 0.11). A weak correlation between age of the FFPE tissue and DNA yield (*r* = 0.337, *p* < 0.01) was observed, but not RNA yield (*r* = − 0.10, *p* = 0.928). Furthermore, there was no significant correlation between percentage of tumor cells or Gleason scores with DNA/RNA yield, A260/A280 ratios, RIN- or C_T_-values (data not shown). A multivariate regression analyses confirmed that tumor length was the main determinant for both RNA and DNA yield, for each additional millimeter tumor the quantity of RNA/DNA increased with 0.53 ng/μl (95% CI: 0.18–0.88) and 0.24 ng/μl (95% CI: 0.11–0.37), respectively. However, an association between tumor length and RIN-values was not found (β = 0.008, 95% CI: -0.03 – 0.05). Age of the FFPE tissue was associated with DNA yield (β = 0.67, 95% CI: 0.35–0.98), and Gleason score (β = − 0.57, 95% CI: -1.08 - -0.06).

**Table 5:** Correlation between tumor length (mm) and RNA quantity and quality

	Tumor length (mm)	RNA Yield	RNA A260/A280	RNA RIN-value	RNA C_T_-value
Tumor length (mm)	1.0	0.45**	0.14	0.37**	−0.25*
RNA Yield	–	1.0	−0.23*	0.19	−0.04
RNA A260/A280	–	–	1.0	−0.01	−0.51**
RNA RIN-value	–	–	–	1.0	−0.29*
RNA C_T_-value	–	–	–	–	1.0

* Significant correlation *p <* 0.05. ** Significant correlation *p <* 0.001 using Pearson Correlation

**Table 6:** Correlation between tumor length (mm) and DNA quantity and quality

	Tumor length (mm)	DNA Yield	DNA A260/A280	DNA C_T_-value
Tumor length (mm)	1.0	0.45**	0.16	−0.13
DNA Yield	–	1.0	0.09	−0.31*
DNA A260/A280	–	–	1.0	−0.11
DNA C_T_-value	–	–	–	1.0

* Significant correlation *p <* 0.05. ** Significant correlation *p <* 0.001 using Pearson Correlation

## Discussion

### Assessment of nucleic acids extraction kits

We compared the quality and quantity of nucleic acid extraction in five commercially available extraction kits, using prostate needle biopsies with normal histology, assuming that normal tissue is less heterogeneous than malignant tissue, thus reducing the risk of biased comparison. We found that RNA extraction with RNeasy® had higher yields of RNA, purity (A260/A280) and RIN-values. Furthermore, the agreement in RNA yield and quality (A260/A280 and RIN-values) between the two compared kits was low, with consistently lower yields and purities in the High Pure kit. RNA of high quality is essential when performing molecular studies, and thus even small differences in purity and RIN-values are important to consider when choosing extraction kit. Based on the results in this trial, the RNeasy® kit would be preferred for extraction of high quality RNA from FFPE prostate biopsies.

In a second trial, the RNeasy® FFPE kit was compared to the AllPrep® DNA/RNA FFPE kit. The two kits produced similar yields and purity of the extracted RNA, however, higher RIN-values were found in samples extracted with the RNeasy® kit. Even though it would typically be preferable to extract both DNA and RNA from the same tissue section, the consistently lower quality of RNA produced with the AllPrep® kit suggests that using two separate extraction kits for DNA and RNA would be preferable if high quality RNA is desired.

A possible explanation for the kit-to-kit variations in RNA quantity and quality could be the conditions recommended for the proteinase K digestion. The use of proteinase K digestion is necessary to release RNA from the meshwork of cross-linked proteins and nucleic acids, and it has also been shown to play an important role in the extraction of longer stretches of nucleic acids (>200 bp) [14, 19, 20]. However, the proteinase K digestion does not attack the methylene bridges that forms the crosslinks [3]. The RNeasy® kit uses a proteinase K treatment for 15 min at 56 °C followed by 15 min at 80 °C, the latter being critical for reversal of crosslinks introduced by formalin fixation. The High Pure kit on the other hand recommends a proteinase K digestion for 3 h at 55 °C. It is therefore possible that the digestion process in the High Pure protocol is less efficient in breaking the crosslinks in the tissue, thus resulting in less RNA yield, which is also more fragmented than samples extracted with the RNeasy® kit.

In a third trial, we compared the two DNA extraction kits QIAamp® DNA FFPE Tissue kit and High Pure FFPET DNA Isolation kit, and found similar DNA yields, although with higher purity (A260/A280) in the QIAamp® kit. One sample extracted with the QIAamp® kit had a high A260/A280 ratio (2.66), which is probably due to a RNA contamination within this sample. Based on these data, we chose the QIAamp® kit for comparison with the AllPrep® DNA/RNA FFPE kit. In this final trial, similar DNA yields in samples extracted with the two kits were found, although once again with higher purity (A260/A280) in the QIAamp®. Thus, our analyses suggest the QIAamp® kit as the best performing DNA extraction kit from FFPE prostate biopsies.

### Assessment of DNA/RNA quantity and quality from archival biopsies

Performing molecular studies on archival FFPE needle biopsy material have potential limitations. Prostate needle biopsies are often performed using an 18-gauge needle, yielding a biopsy which is roughly 1 mm in diameter and 12–15 mm long. Sectioning such a biopsy would in theory yield 100 sections of 10 μm. However, the biopsy has typically been sectioned previously as a part of clinical evaluation, and thus only a limited amount of tissue with representative disease histology is available for research studies. Furthermore, according to Swedish laws one must make sure that there is always representative cancer tissue left after sections are taken for research studies. Thus, in practice, it might be possible to take four to five 10 μm sections for research studies. The aim of this second part of the study was thus to investigate if it is possible to extract nucleic acids from archival FFPE prostate biopsies using one 10 μm section of tissue. We also wanted to investigate the quantity and quality of the nucleic acids extracted from the biopsies in order to investigate which type of molecular studies could be performed.

Based on the results from the assessment of nucleic acid extraction kits, nucleic acids were extracted using the QIAamp® DNA FFPE Tissue kit and RNeasy® FFPE kit. DNA was successfully extracted from all 84 biopsies with a mean yield of 3.7 ng/μl and a purity of 1.7. One DNA extract had a high A260/A280 ratio (5.3), indicating a RNA contamination of this sample. RNA was also successfully extracted from all 84 samples, with a mean yield of 10.6 ng/μl. The purity of the extracted RNA had a mean value of 1.55 and the RIN-value a mean of 2.3. The expression of 18S5 RNA showed high variability between samples, indicating that this is not an optimal endogenous control gene for prostate tissues. The amount of tumor (in millimeters) had the strongest association with both quantity and quality of the nucleic acids extracted from the biopsies within this study, which is consistent with a previous study showing an association between tumor amount, measured by millimeter tumor of a renal biopsy, and purity of RNA (A260/A280) [21]. Our results indicated no correlation between age of the FFPE specimen and neither RNA yield or integrity of the extracted RNA. This result has been supported by several previous studies [10, 21, 22], although others have seen an association between age of the tissue and RNA quality, but not yield [23]. However, we did find a correlation between age of the FFPE specimen and DNA yield in the present study, which has also been shown previously by Carrick et al.*,* who observed a significant association between DNA yield and quality and storage time [24]. Nucleic acids extracted from FFPE samples are generally more degraded than samples from fresh frozen material, resulting in reduced qPCR amplification of DNA and RNA targets larger than 200 base pairs (bp) [25–27]. In the present study, a 90 bp amplicon of 18S5 rRNA was amplified with success (C_T_ < 38) in 83 of 84 (98.8%) RNA samples and 81 of 84 (96.4%) DNA samples. The RNA sample that was not amplified had the lowest purity of all samples (A260/A280 = 0.7), however for the DNA samples there was no apparent reason to why the amplification failed. These results indicate that archival FFPE prostate biopsy tissues are suitable for PCR amplification even though the small quantity and poor quality of the isolated nucleic acids.

In order to gain new insights to pathological processes, quantitative measures of mRNA levels and DNA analyses investigating for example mutations, polymorphisms and epigenetic changes have become essential. It is recommended that only high-quality DNA and RNA are used for these analyses. In some instances, however, such as in studies of biomarkers of prostate cancer mortality, high quality samples may not be available. Typically using low-quality samples introduces bias toward the null, because the degradation of samples entails random variability that is similar across comparison groups. Therefore, precision and power is impaired, and the risk of false negative associations, but not false positive, increases.

Many studies have investigated the effect of poor RNA quality (RIN-value) and DNA quality, using techniques such as qPCR, microarrays and NGS [10, 12, 23, 24, 26, 28–35]. RIN-values are considered the most powerful predictor of microarray quality [30]. Although moderately degraded RNA could yield acceptable microarray results, extensively degraded samples such as those obtained in the present study, should usually be excluded from analyses. Only samples with RIN-values ≥7 are normally recommended for microarray studies [36]. However, the cDNA-mediated Annealing, Selection, extension and Ligation (DASL) assay have been shown to tolerate input RNA degraded to an average of 100–200 bp, while still yielding robust results [37]. This technology has successfully been used to profile archival FFPE prostatic tissues previously, including by our group [38–41]. Achieving information on single nucleotide polymorphisms (SNPs) in FFPE DNA has been an issue due to low quantity and quality of the DNA. However, studies comparing results obtained from fresh frozen and FFPE tissues have shown a high concordance for some SNP arrays [42, 43], and new platforms have allowed generation of reliable results even from small quantities of FFPE DNA [44]. NGS is another technology used for both large-scale and targeted analysis of both DNA and RNA samples, and it has been shown that the success rate of the technique is not associated with storage time of the FFPE tissue, not even for samples stored up to 32 years [24]. Furthermore, a good correlation of NGS data has been found between fresh frozen and FFPE tissues, even though a higher sequencing coverage (× 80) is recommended for FFPE tissues [23, 31–35]. Measuring gene expression levels in FFPE samples using qPCR has been suggested to be more robust than through microarrays [26]. This suggestion is strengthened by findings showing that RIN-values cannot be used in order to predict performance of the qPCR [10, 26, 45, 46]. Amplification of products > 400 bp using qPCR is strongly dependent on good RNA quality (RIN > 5), although if smaller amplicons (70–250 bp) are investigated, the results are more or less independent of RNA quality [12]. In the past it has been problematic to perform large scale qPCR analyses on FFPE samples due to high C_T_-values and limited concentrations of the extracts [47, 48], however techniques such as TaqMan® PreAmp has addressed this challenge faced by researchers working with small samples such as needle biopsies, from which only limited amounts of RNA could be extracted. The simple process includes pre-amplification of as little as one ng of cDNA, and enables the user to perform qPCR amplification of up to 384 target genes per pre-amplification reaction on TaqMan® low-density arrays [49]. Targeted qPCR and pyrosequencing can also be used to provide reliable data when analyzing FFPE DNA samples [50], and the methods can overcome the limits of using FFPE DNA by designing assays specific for small DNA fragments and improving the PCR protocol for low DNA inputs [51, 52].

An advantage with our study is that the extraction kits have been compared using serial sections of the same biopsies, which decreases the risk of differences between the kits due to variations in the tissue histology. Furthermore, the extraction kits were tested on biopsies with normal histology, which further decreases tissue heterogeneity. The best performing extraction kits for DNA and RNA was subsequently used for extraction of nucleic acids on 84 biopsies with malignant histology, yielding similar quantities and quality of the nucleic acids as in the normal biopsies. The study has a number of limitations. 1) The limited number of nucleic acid extraction kits tested. Many other kits for extraction of nucleic acids from FFPE tissues exist on the market, with the potential to perform better than the kits used in this study. 2) The small number of comparisons performed for each extraction kit (*n* = 10). Further studies on larger tissue materials are needed in order to validate the results found within the present study. 3) Even though the tissue sections used for comparisons of extraction kits were cut immediately adjacent to one another, there could still have been variations in the tissue area that influenced the DNA/RNA yield. 4) Due to the limited amount of tissue available in a prostate biopsy, extractions could not be performed in duplicates from the same biopsy. Furthermore, two different operators performed the extraction of nucleic acids with the kits to be compared, which could have affected the outcome due to inter-operator variability [15].

## Conclusions

To conclude, our results indicate that it is possible to utilize archival FFPE prostate biopsies in biomarker studies for PCa, and that the choice of extraction kit could be of importance to obtain high quality DNA and RNA from FFPE tissues.

## Abbreviations

C_T_: Cycle threshold
FFPE: Formalin-fixed paraffin-embedded
NGS: Next generation sequencing
PCa: Prostate Cancer
RIN: RNA Integrity Number
SNP: Single Nucleotide Polymorphism

## References

[1] Ferlay J, Steliarova-Foucher E, Lortet-Tieulent J, Rosso S, Coebergh JW, Comber H, Forman D, Bray F (2013). Cancer incidence and mortality patterns in Europe: estimates for 40 countries in 2012. Eur J Cancer.

[2] Fox CH, Johnson FB, Whiting J, Roller PP (1985). Formaldehyde fixation. J Histochem Cytochem.

[3] Masuda N, Ohnishi T, Kawamoto S, Monden M, Okubo K (1999). Analysis of chemical modification of RNA from formalin-fixed samples and optimization of molecular biology applications for such samples. Nucleic Acids Res.

[4] Douglas MP, Rogers SO (1998). DNA damage caused by common cytological fixatives. Mutat Res.

[5] McGhee JD, von Hippel PH (1975). Formaldehyde as a probe of DNA structure. I. Reaction with exocyclic amino groups of DNA bases. Biochemistry.

[6] Srinivasan M, Sedmak D, Jewell S (2002). Effect of fixatives and tissue processing on the content and integrity of nucleic acids. Am J Pathol.

[7] Chung JY, Braunschweig T, Hewitt SM (2006). Optimization of recovery of RNA from formalin-fixed, paraffin-embedded tissue. Diagn Mol Pathol.

[8] Doleshal M, Magotra AA, Choudhury B, Cannon BD, Labourier E, Szafranska AE (2008). Evaluation and validation of total RNA extraction methods for microRNA expression analyses in formalin-fixed, paraffin-embedded tissues. J Mol Diagn.

[9] Okello JB, Zurek J, Devault AM, Kuch M, Okwi AL, Sewankambo NK, Bimenya GS, Poinar D, Poinar HN (2010). Comparison of methods in the recovery of nucleic acids from archival formalin-fixed paraffin-embedded autopsy tissues. Anal Biochem.

[10] von Ahlfen S, Missel A, Bendrat K, Schlumpberger M (2007). Determinants of RNA quality from FFPE samples. PLoS One.

[11] Miyatake Y, Ikeda H, Michimata R, Koizumi S, Ishizu A, Nishimura N, Yoshiki T (2004). Differential modulation of gene expression among rat tissues with warm ischemia. Exp Mol Pathol.

[12] Fleige S, Walf V, Huch S, Prgomet C, Sehm J, Pfaffl MW (2006). Comparison of relative mRNA quantification models and the impact of RNA integrity in quantitative real-time RT-PCR. Biotechnol Lett.

[13] Janecka A, Adamczyk A, Gasinska A (2015). Comparison of eight commercially available kits for DNA extraction from formalin-fixed paraffin-embedded tissues. Anal Biochem.

[14] Kocjan BJ, Hosnjak L, Poljak M (2015). Commercially available kits for manual and automatic extraction of nucleic acids from formalin-fixed, paraffin-embedded (FFPE) tissues. Acta Dermatovenerol Alp Pannonica Adriat.

[15] Bonin S, Hlubek F, Benhattar J, Denkert C, Dietel M, Fernandez PL, Hofler G, Kothmaier H, Kruslin B, Mazzanti CM (2010). Multicentre validation study of nucleic acids extraction from FFPE tissues. Virchows Arch.

[16] Potluri K, Mahas A, Kent MN, Naik S, Markey M (2015). Genomic DNA extraction methods using formalin-fixed paraffin-embedded tissue. Anal Biochem.

[17] Schroeder A, Mueller O, Stocker S, Salowsky R, Leiber M, Gassmann M, Lightfoot S, Menzel W, Granzow M, Ragg T (2006). The RIN: an RNA integrity number for assigning integrity values to RNA measurements. BMC Mol Biol.

[18] Ludbrook J (1997). Comparing methods of measurements. Clin Exp Pharmacol Physiol.

[19] Kraus E, Kiltz HH, Femfert UF (1976). The specificity of proteinase K against oxidized insulin B chain. Hoppe Seylers Z Physiol Chem.

[20] Huijsmans CJ, Damen J, van der Linden JC, Savelkoul PH, Hermans MH (2010). Comparative analysis of four methods to extract DNA from paraffin-embedded tissues: effect on downstream molecular applications. BMC Res Notes.

[21] Reich HN, Landolt-Marticorena C, Boutros PC, John R, Wither J, Fortin PR, Yang S, Scholey JW, Herzenberg AM (2011). Molecular markers of injury in kidney biopsy specimens of patients with lupus nephritis. J Mol Diagn.

[22] Patel PG, Selvarajah S, Boursalie S, How NE, Ejdelman J, Guerard KP, Bartlett JM, Lapointe J, Park PC, Okello JB, et al. Preparation of formalin-fixed paraffin-embedded tissue cores for both RNA and DNA extraction. J Vis Exp. 2016;(114):54299. 10.3791/54299 PMC5091935 27583817

[23] Hedegaard J, Thorsen K, Lund MK, Hein AM, Hamilton-Dutoit SJ, Vang S, Nordentoft I, Birkenkamp-Demtroder K, Kruhoffer M, Hager H (2014). Next-generation sequencing of RNA and DNA isolated from paired fresh-frozen and formalin-fixed paraffin-embedded samples of human cancer and normal tissue. PLoS One.

[24] Carrick DM, Mehaffey MG, Sachs MC, Altekruse S, Camalier C, Chuaqui R, Cozen W, Das B, Hernandez BY, Lih CJ (2015). Robustness of next generation sequencing on older formalin-fixed paraffin-embedded tissue. PLoS One.

[25] Klopfleisch R, Weiss AT, Gruber AD (2011). Excavation of a buried treasure--DNA, mRNA, miRNA and protein analysis in formalin fixed, paraffin embedded tissues. Histol Histopathol.

[26] Kashofer K, Viertler C, Pichler M, Zatloukal K (2013). Quality control of RNA preservation and extraction from paraffin-embedded tissue: implications for RT-PCR and microarray analysis. PLoS One.

[27] Koopmans M, Monroe SS, Coffield LM, Zaki SR (1993). Optimization of extraction and PCR amplification of RNA extracts from paraffin-embedded tissue in different fixatives. J Virol Methods.

[28] Opitz L, Salinas-Riester G, Grade M, Jung K, Jo P, Emons G, Ghadimi BM, Beissbarth T, Gaedcke J (2010). Impact of RNA degradation on gene expression profiling. BMC Med Genet.

[29] Strand C, Enell J, Hedenfalk I, Ferno M (2007). RNA quality in frozen breast cancer samples and the influence on gene expression analysis--a comparison of three evaluation methods using microcapillary electrophoresis traces. BMC Mol Biol.

[30] Kiewe P, Gueller S, Komor M, Stroux A, Thiel E, Hofmann WK (2009). Prediction of qualitative outcome of oligonucleotide microarray hybridization by measurement of RNA integrity using the 2100 Bioanalyzer capillary electrophoresis system. Ann Hematol.

[31] Kerick M, Isau M, Timmermann B, Sultmann H, Herwig R, Krobitsch S, Schaefer G, Verdorfer I, Bartsch G, Klocker H (2011). Targeted high throughput sequencing in clinical cancer settings: formaldehyde fixed-paraffin embedded (FFPE) tumor tissues, input amount and tumor heterogeneity. BMC Med Genet.

[32] Wood HM, Belvedere O, Conway C, Daly C, Chalkley R, Bickerdike M, McKinley C, Egan P, Ross L, Hayward B (2010). Using next-generation sequencing for high resolution multiplex analysis of copy number variation from nanogram quantities of DNA from formalin-fixed paraffin-embedded specimens. Nucleic Acids Res.

[33] Schweiger MR, Kerick M, Timmermann B, Albrecht MW, Borodina T, Parkhomchuk D, Zatloukal K, Lehrach H (2009). Genome-wide massively parallel sequencing of formaldehyde fixed-paraffin embedded (FFPE) tumor tissues for copy-number- and mutation-analysis. PLoS One.

[34] Li P, Conley A, Zhang H, Kim HL (2014). Whole-transcriptome profiling of formalin-fixed, paraffin-embedded renal cell carcinoma by RNA-seq. BMC Genomics.

[35] Graw S, Meier R, Minn K, Bloomer C, Godwin AK, Fridley B, Vlad A, Beyerlein P, Chien J (2015). Robust gene expression and mutation analyses of RNA-sequencing of formalin-fixed diagnostic tumor samples. Sci Rep.

[36] Raman T, O'Connor TP, Hackett NR, Wang W, Harvey BG, Attiyeh MA, Dang DT, Teater M, Crystal RG (2009). Quality control in microarray assessment of gene expression in human airway epithelium. BMC Genomics.

[37] Fan JB, Yeakley JM, Bibikova M, Chudin E, Wickham E, Chen J, Doucet D, Rigault P, Zhang B, Shen R (2004). A versatile assay for high-throughput gene expression profiling on universal array matrices. Genome Res.

[38] Li HR, Wang-Rodriguez J, Nair TM, Yeakley JM, Kwon YS, Bibikova M, Zheng C, Zhou L, Zhang K, Downs T (2006). Two-dimensional transcriptome profiling: identification of messenger RNA isoform signatures in prostate cancer from archived paraffin-embedded cancer specimens. Cancer Res.

[39] Bibikova M, Chudin E, Arsanjani A, Zhou L, Garcia EW, Modder J, Kostelec M, Barker D, Downs T, Fan JB (2007). Expression signatures that correlated with Gleason score and relapse in prostate cancer. Genomics.

[40] Nakagawa T, Kollmeyer TM, Morlan BW, Anderson SK, Bergstralh EJ, Davis BJ, Asmann YW, Klee GG, Ballman KV, Jenkins RB (2008). A tissue biomarker panel predicting systemic progression after PSA recurrence post-definitive prostate cancer therapy. PLoS One.

[41] Setlur SR, Mertz KD, Hoshida Y, Demichelis F, Lupien M, Perner S, Sboner A, Pawitan Y, Andren O, Johnson LA (2008). Estrogen-dependent signaling in a molecularly distinct subclass of aggressive prostate cancer. J Natl Cancer Inst.

[42] Tuefferd M, De Bondt A, Van Den Wyngaert I, Talloen W, Verbeke T, Carvalho B, Clevert DA, Alifano M, Raghavan N, Amaratunga D (2008). Genome-wide copy number alterations detection in fresh frozen and matched FFPE samples using SNP 6.0 arrays. Genes Chromosomes Cancer.

[43] Oosting J, Lips EH, van Eijk R, Eilers PH, Szuhai K, Wijmenga C, Morreau H, van Wezel T (2007). High-resolution copy number analysis of paraffin-embedded archival tissue using SNP BeadArrays. Genome Res.

[44] Fraser M, Sabelnykova VY, Yamaguchi TN, Heisler LE, Livingstone J, Huang V, Shiah YJ, Yousif F, Lin X, Masella AP (2017). Genomic hallmarks of localized, non-indolent prostate cancer. Nature.

[45] Abramovitz M, Ordanic-Kodani M, Wang Y, Li Z, Catzavelos C, Bouzyk M, Sledge GW, Moreno CS, Leyland-Jones B (2008). Optimization of RNA extraction from FFPE tissues for expression profiling in the DASL assay. BioTechniques.

[46] Ribeiro-Silva A, Zhang H, Jeffrey SS (2007). RNA extraction from ten year old formalin-fixed paraffin-embedded breast cancer samples: a comparison of column purification and magnetic bead-based technologies. BMC Mol Biol.

[47] Cohen CD, Grone HJ, Grone EF, Nelson PJ, Schlondorff D, Kretzler M (2002). Laser microdissection and gene expression analysis on formaldehyde-fixed archival tissue. Kidney Int.

[48] Abrahamsen HN, Steiniche T, Nexo E, Hamilton-Dutoit SJ, Sorensen BS (2003). Towards quantitative mRNA analysis in paraffin-embedded tissues using real-time reverse transcriptase-polymerase chain reaction: a methodological study on lymph nodes from melanoma patients. J Mol Diagn.

[49] Li J, Smyth P, Cahill S, Denning K, Flavin R, Aherne S, Pirotta M, Guenther SM, O'Leary JJ, Sheils O (2008). Improved RNA quality and TaqMan pre-amplification method (PreAmp) to enhance expression analysis from formalin fixed paraffin embedded (FFPE) materials. BMC Biotechnol.

[50] Leong KJ, James J, Wen K, Taniere P, Morton DG, Bach SP, Matthews GM (2013). Impact of tissue processing, archiving and enrichment techniques on DNA methylation yield in rectal carcinoma. Exp Mol Pathol.

[51] Dietrich D, Uhl B, Sailer V, Holmes EE, Jung M, Meller S, Kristiansen G (2013). Improved PCR performance using template DNA from formalin-fixed and paraffin-embedded tissues by overcoming PCR inhibition. PLoS One.

[52] Doyle B, O'Riain C, Appleton K (2011). Pyrosequencing of DNA extracted from formalin-fixed paraffin-embedded tissue. Methods Mol Biol.

